# Tissue Kallikrein Protects Rat Prostate against the Inflammatory Damage in a Chronic Autoimmune Prostatitis Model via Restoring Endothelial Function in a Bradykinin Receptor B2-Dependent Way

**DOI:** 10.1155/2022/1247806

**Published:** 2022-02-01

**Authors:** Mengyang Zhang, Dongxu Lin, Changcheng Luo, Pengyu Wei, Kai Cui, Zhong Chen

**Affiliations:** ^1^Department of Urology, Tongji Hospital, Tongji Medical College, Huazhong University of Science and Technology, Wuhan, 430030 Hubei, China; ^2^Institute of Urology, Tongji Hospital, Tongji Medical College, Huazhong University of Science and Technology, Wuhan, 430030 Hubei, China

## Abstract

**Objective:**

The aim of this study was to investigate whether tissue kallikrein (KLK1) can protect the prostate from inflammatory damage and the mechanism involved in it.

**Methods:**

A total of 50 male Wistar rats were used in this study. Initially, 20 rats were sacrificed to obtain the prostate antigen to induce experimental autoimmune prostatitis (EAP), and the remaining 30 rats were randomly divided into 5 experimental groups (normal control group (NC group), NC+KLK1 group (NCK group), EAP group, EAP+KLK1 group (EAPK group), and EAP+KLK1+HOE140 group (EAPKH group); *n* = 6). It should be explained that KLK1 mainly exerts its biological effects through bradykinin, and HOE140 is a potent and selective bradykinin receptor B2 (BDKRB2) antagonist. EAP was induced by intradermal injection of 15 mg/ml prostate antigen and complete Freund's adjuvant on days 0, 14, and 28. KLK1 was injected via tail vein at a dose of 1.5 × 10^−3^ PAN U/kg once a day, and HOE140 was administered by intraperitoneal injection at 20 *μ*g/kg once every two days. Rats were sacrificed on day 42. The RNA and protein of the rat prostate were extracted to analyze the expression differences of KLK1, as well as the inflammation-, fibrosis-, and oxidative stress-related genes. The inflammatory cell infiltration and microvessel density of the prostate were also analyzed by pathological examination. In addition, pathological analysis was performed on prostate samples from patients undergoing benign prostate hyperplasia (BPH) surgery.

**Results:**

The expression of KLK1 in the prostate decreased in the EAP group as well as BPH patients with obvious inflammation. KLK1 administration significantly inhibited inflammatory cell infiltration and reduced the production of inflammatory cytokines in the EAPK group. Prostate samples from the EAP group showed increased infiltration of T cells and macrophages, as well as gland atrophy, hypoxia, fibrosis, and angiogenesis. KLK1 administration upregulated endothelial nitric oxide synthase (eNOS) expression and suppressed oxidative stress, as well as transforming growth factor *β*1 (TGF-*β*) signaling pathways and the proangiogenic vascular endothelial growth factor (VEGF) in the EAPK group. However, in the EAPKH group in which HOE140 blocked BDKRB2, the beneficial effects of KLK1 were all cancelled. In addition, KLK1 intervention in normal rats had no obvious side effects.

**Conclusion:**

The KLK1 expression is inhibited in the inflamed prostates of humans and rats. Exogenous KLK1 restored endothelial function via a BDKRB2-dependent way and then played a role in improving microcirculation and exerted anti-inflammatory, antifibrotic, and antioxidative stress effects in the rat chronic-inflamed prostate.

## 1. Introduction

BPH is a common and bothersome benign proliferative disease and has a significant impact on the quality of life for middle-aged and older men. The prevalence of BPH rises with age. Approximately 40% of 50-year-old men and 90% of 90-year-old men suffer BPH [1]. With age, a variety of pathogenic factors have jointly caused the progressive hyperplasia of prostatic glandular epithelial and stromal cells and other pathological changes, which further leads to prostate enlargement and bladder outflow obstruction and eventually leads to LUTS, such as urinary hesitancy, frequency incontinence, pain, and nocturia. We have previously reported the protective effect of KLK1 on the rodent aging prostate [2]. Inflammation is one of the important mechanisms of BPH progression discovered in recent years. Studies have shown that inflammation is not just an occasional concomitant, but instead, plays a key role in its etiology [3–7]. In this article, we will report the effect of KLK1 on the inflammation mechanism in BPH/LUTS by using an EAP rat model.

Evidence from clinical research shows that prostatic inflammation is more common in men with BPH/LUTS and correlates with symptom severity and risk for progression [8, 9]. A study with pathological analysis of surgical samples from prostate cancer patients shows that chronic prostatitis tissues have a larger stromal volume than normal prostate tissues [10]. In laboratory research, a variety of inflammatory mediators that promote local angiogenesis and the proliferation of prostate epithelial or stromal cells have been discovered [3, 11],, and BPH-related pathological manifestations have also been observed in an EAP rat model [12]. The administration of anti-inflammatory drugs in chronic prostatitis rats induced by estradiol prevents inflammation infiltration and decreases the stroma-to-epithelium ratio [7, 13]. Additionally, many animal experiments show that inflammation also induces chronic pelvic pain, oxidative stress, angiogenesis, stroma remodeling, and collagen deposition in the prostate [12, 14–18]. In conclusion, inflammation may drive nociceptive signaling, hypoxia, and hyperplastic growth and fibrosis in BPH/LUTS.

The kallikrein-kinin system is composed of kininogens, kallikrein, kinin peptides, kinin-degrading enzymes, and kinin receptors, and its biological function is primarily mediated by kinin peptides and subsequent kinin receptor activation [19]. The system is an endogenous multiprotein metabolic cascade that is implicated in a plethora of biological processes and homeostasis [20]. KLK1, a key component of the system, participates in the regulation of microcirculation, blood pressure, and blood flow via processing LMWK to release the BK [21]. BK binds to the bradykinin receptors B1 and B2 to activate several downstream targets such as NO, cGMP, prostacyclin, and cAMP [20]. The B2 receptor is a constitutively expressed G protein-coupled receptor and has been proved to exhibit a wide spectrum of beneficial effects, whereas the B1 receptor is expressed at very low levels under normal conditions [19]. Many studies have proved that the KLK1 replenishment therapy is beneficial to many diseases involving poor microcirculation and could exhibit anti-inflammatory, antifibrotic, and antioxidative effects to ameliorate end-organ dysfunction [19, 21]. Previously, we suggested that KLK1 could attenuate age-related prostate and penile damage in aged transgenic rats harboring the human KLK1 gene by reducing fibrosis and oxidative stress via regulation of related signaling pathways [2, 22, 23]. And we also found the protective effect of KLK1 on the corpus cavernosum endothelial cells in a rat model of hyperhomocysteinemia [24]. However, whether KLK1 can protect the prostate from inflammatory damage and thus help inhibit the progression of BPH/LUTS remains unclear.

## 2. Materials and Methods

### 2.1. Experimental Animals

Animal experiments of this study were approved by the Ethics Committee and Academic Administration Committee of Tongji Hospital, Tongji Medical College, Huazhong University of Science and Technology (Wuhan, China), with ethics number TJH-202101005. All experiment procedures were in strict accordance with the *Guide for the Care and Use of Laboratory Animals* published by the US National Institutes of Health. A total of 50 male 8-week-old Wistar rats were purchased from the Vital River Laboratory Animal Technology Co. Ltd. (Beijing, China). All rats were individually housed in a SPF facility with laminar flow, maintained at 20°C ± 1°C and 50% ± 10% relative humidity with a 12 h light/12 h dark photoperiod, and bred by professional breeders.

### 2.2. Analysis of Samples from BPH Patients

All retrospective clinical data analyses and prostate specimen collection were performed after obtaining informed consent from all patients and approval of the Ethics Committee and Academic Administration Committee of Tongji Hospital (ethics number: TJ-IRB20211113). The H&E-stained sections, Masson-stained sections, and KLK1 IHC sections involved in this current research were properly preserved sections that were made and used in our previous research [2]. The specific experimental process has been described in the previous article. Formalin-fixed paraffin-embedded prostate tissue blocks were obtained from 47 patients who were diagnosed with BPH pathologically and underwent transurethral prostatectomy in the Tongji Hospital from 2018 to 2019, aged from 48 to 92 years. Patients suspected of having malignant pathological features were excluded.

By examining the H&E sections, we found 6 samples with obvious inflammation infiltration. It should be noted that samples infiltrated with only a single mild inflammatory focus or a small number of scattered inflammatory cells were excluded. We compared the difference in the Masson-positive area ratio and the expression level of KLK1 protein between the 6 samples with obvious inflammation and the remaining 41 samples without obvious inflammation.

### 2.3. Animal Grouping and Drug Administration

All rats were untreated until sexual maturity at 12 weeks old. Then, they were randomly divided into 6 groups, including 5 experimental groups with 6 rats in each group and another group of 20 rats sacrificed to obtain the prostate antigen. According to different interventions, the 5 experimental groups were named as the normal control group (NC group), NC+KLK1 group (NCK group), experimental autoimmune prostatitis group (EAP group), EAP+KLK1 group (EAPK group), and EAP+KLK1+HOE140 group (EAPKH group).

**Figure 1:**
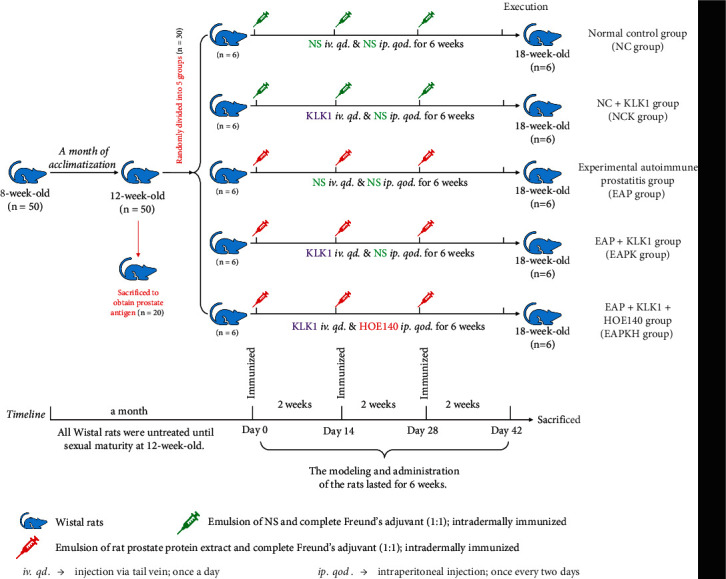
Animal grouping and flowchart.

The method of inducing EAP was based on previous studies [12, 16]. In our current research, 20 rats were sacrificed by cervical dislocation, and the prostate tissues were obtained and homogenized in 0.5% Triton X-100 (catalog no. 20107ES76, Yeasen Biotechnology, China); the homogenates were maintained in an ice water bath. They were then centrifuged at 10,000*g* for 30 min at 4°C, and the supernatants were separated. The protein concentrations in the supernatants were assayed by the BCA protein quantification assay kit (catalog no. AR1189A, Boster Biological Technology, China) and diluted to concentrations of 15 mg/ml with 0.1 mol/l PBS buffer. Equal volumes of rat prostate protein extract and complete Freund's adjuvant (catalog no. F5881, Sigma-Aldrich, USA) were emulsified for immunization. The rats in the EAP group, EAPK group, and EAPKH group were anesthetized by the intraperitoneal injection of pentobarbital (40 mg/kg). Then, each rat was intradermally immunized in the hind footpad and the base of the tail with a total of 1.0 ml of emulsion. The rats in the NC group and NCK group were immunized with NS emulsified in complete Freund's adjuvant. All rats received immunization on days 0, 14, and 28 of the experimental schedule and were sacrificed on day 42. The ventral lobes of the prostate were quickly removed, 1/3 of the bilateral ventral lobes were fixed with 4% paraformaldehyde for 24 h and then embedded in paraffin for histologic studies, and the remaining tissues were frozen at -80°C. The specific experimental process is shown in Figure 1.

The KLK1 used in this study was a drug that had been approved by the Chinese regulatory drug agency called Kailikang® (Techpool Bio-Pharma, China), which was composed of human urinary KLK1 and adjuvants. Kailikang® was dissolved in NS at 1.5 × 10^−3^ PAN U/ml. The rats in the NCK group, EAPK group, and EAPKH group were injected with KLK1 via tail vein at a dose of 1.5 × 10^−3^ PAN U/kg once a day. HOE140 (catalog no. 3014, R&D Systems, USA) is a potent and specific peptide antagonist of bradykinin B2 receptor [25]. HOE140 was first dissolved in sterile distilled water and stored in aliquots at -80°C and then diluted with normal saline before administration to rats of the EAPKH group at a dose of 20 *μ*g/kg once every two days. All rats were given drugs or an equal volume of the vehicle in the same way.

### 2.4. RNA Isolation and QRT-PCR

Total RNA was extracted from frozen tissues with the TRIzol reagent (catalog no. 15596018, Invitrogen, USA) and quantitated at 260/280 nm using a NanoDrop ND-1000 spectrophotometer (NanoDrop Technologies, USA). 1 *μ*g of each total RNA sample was reverse transcribed into cDNA using a PrimeScript™ RT Master Mix (catalog no. RR036A, TaKaRa, China). QRT-PCR was performed to determine the mRNA levels of genes of interest based on TB Green® Premix Ex Taq™ II (catalog no. RR82LR, TaKaRa, China) using a QuantStudio™ 6 Flex Real-Time PCR System (Thermo Fisher, USA). Primers were provided by the Tsingke Biotechnology (China). The mRNA expression levels of the examined genes were normalized to that of *β*-actin; the relative mRNA expression levels were calculated using the 2^−*ΔΔ*Ct^ method. All samples were independently repeated for analysis three times.

### 2.5. Western Blot Analysis

Rat prostate tissue was lysed with RIPA protein extraction buffer (catalog no. AR0105, Boster Biological Technology, China) containing phosphatase and protease inhibitors (catalog no. AR1182-1, Boster Biological Technology, China). Equal quantities of protein were resolved by electrophoresis on SOD SDS polyacrylamide gels and transferred onto PVDF membranes (Millipore, USA) by a voltage gradient. After being blocked with 5% BSA for 1 hour at room temperature, the membranes were incubated overnight at 4°C with primary antibodies and then incubated with HRP-conjugated secondary antibodies for 1 h. After washing three times, the bands were analyzed using an enhanced chemiluminescence detection system (Pierce, Thermo Fisher Scientific). The primary antibodies against KLK1 (catalog no. 32443) and *β*-actin (catalog no. 21800) were purchased from the Signalway Antibody (USA). The primary antibodies against Collagen Type I (catalog no. 14695-1-AP), Collagen Type III (catalog no. 22734-1-AP), TGF-*β* (catalog no. 21898-1-AP), RhoA (catalog no. 10749-1-AP), ROCK1 (catalog no. 21850-1-AP), HIF-1*α* (catalog no. 20960-1-AP), VEGF (catalog no. 19003-1-AP), and eNOS (catalog no. 27120-1-AP) were purchased from the Proteintech (China). The optical density of the band was analyzed by ImageJ software (NIH, USA). The data were normalized using *β*-actin as an internal control. All samples were analyzed independently via three repetitions, and the mean values were determined.

### 2.6. Histological Examinations

#### 2.6.1. H&E and Masson's Trichrome Staining

The 4% paraformaldehyde-fixed prostate tissue samples were subjected to paraffin embedding and sectioned at a thickness of 5 *μ*m. The tissue sections were stained with H&E Staining Kit (catalog No. G1120, Solarbio, China) and Masson's Trichrome Stain Kit (catalog no. G1340, Solarbio, China) using standard procedures, respectively, and imaged under a light microscope (Olympus, Japan). The severity of inflammation of the rat prostates was assessed by determining the histological score according to the H&E photos. As previously described [26], the pathologic grade was evaluated in 5 random fields at 100x magnification of each section in a double-blind manner. The degree of inflammation was assessed using a score of 0~3: 0, no inflammation; 1, mild but definite perivascular cuffing with mononuclear cells; 2, moderate perivascular cuffing with mononuclear cells; and 3, marked perivascular cuffing, hemorrhage, and numerous mononuclear cells in the parenchyma. In Masson photos, the blue-stained area was mainly composed of collagen components, which was considered to be the positive area of Masson staining in our current research. The Masson-positive area ratio was quantitatively analyzed using ImagePro Plus 6.0 software (Media Cybernetics) in 5 random fields at 100x magnification of each section.

#### 2.6.2. IHC

Sections were incubated overnight at 4°C with antibodies against KLK1, CD3 (catalog no. 17617-1-AP, Proteintech), CD68 (catalog no. 28058-1-AP, Proteintech), and CD34 (catalog no. 14486-1-AP, Proteintech). Sections were then washed three times and incubated with a biotinylated secondary antibody. Finally, antigen-antibody reactions were detected by staining with diaminobenzidine (Beyotime, China). Quantitative analysis was performed to evaluate the AOD of KLK1-positive area using ImagePro Plus 6.0 software. CD3-positive T cells, CD68-positive macrophages, and CD34-positive microvessels were visually counted by the researchers in a double-blind manner. Five random fields at 200x magnification of each section were evaluated.

#### 2.6.3. IF

The sections were washed and incubated with antibodies specific for *α*-SMA (catalog no. 14395-1-AP, Proteintech) overnight at 4°C and then with a secondary antibody for 1 h in the dark at room temperature. The sections were mounted by adding DAPI (Southern Biotech) and examined using a fluorescence microscope (Olympus, Japan). The fluorescence intensity of 5 random fields at 200x magnification of each section was analyzed using ImageJ software.

### 2.7. Analysis of Rat Prostate Cytokine Protein Profile

One rat prostate sample was randomly selected from each of the NC group, EAP group, EAPK group, and EAPKH group, respectively. An equal amount of frozen prostate tissue was powdered under liquid nitrogen and homogenized in PBS with protease inhibitors. The protein concentration of the tissue homogenate was detected by the BCA protein quantification assay kit. Prostate cytokine protein levels were analyzed using Rat Cytokine Array Panel A (catalog no. ARY008, R&D Systems, USA) according to the manufacturer's instructions. Pixel densities of 13 cytokines with visible differences on the 4 developed X-ray films were analyzed using ImageJ software.

### 2.8. Assessment of MDA, SOD, and ROS

The MDA detection kit (catalog no. A003-1-2) and SOD detection kit (catalog no. A001-3-2) were purchased from the Nanjing Jiancheng Bioengineering Institute (China). The assays were performed according to the manufacturer's instructions. All samples were analyzed independently via three repetitions, and the total protein concentrations were detected to normalize the data. The ROS generated in situ in the prostate tissues was assessed using dihydroethidium (catalog no. D7008, Sigma-Aldrich, China), according to the manufacturer's instructions. DAPI was used for staining the nuclei to facilitate cell counting. The stained tissues were visualized using a fluorescence microscope. Relative ROS content = ROS fluorescence intensity/number of cells. Five random fields at 200x magnification of each section were analyzed using ImageJ software.

### 2.9. Statistical Analysis

The results were analyzed using GraphPad Prism 9.0 software (GraphPad Software, USA) and expressed as mean ± SD. Differences between 2 groups were analyzed by two-tailed Student's *t*-test. One-way ANOVA followed by Tukey's multiple comparison test was used to identify differences among multiple groups. In particular, when comparing the data of the 2 types of BPH samples with different levels of inflammation, the medians were displayed, and the Mann-Whitney test was used to identify whether the differences were significant. Statistical differences were regarded as significant when *P* < 0.05 (ns, *P* > 0.05; ^∗^*P* < 0.05; ^∗∗^*P* < 0.01; ^∗∗∗^*P* < 0.001; and ^∗∗∗∗^*P* < 0.0001).

## 3. Results

### 3.1. Downregulation of KLK1 during the Development of Chronic Prostatitis

**Figure 2:**
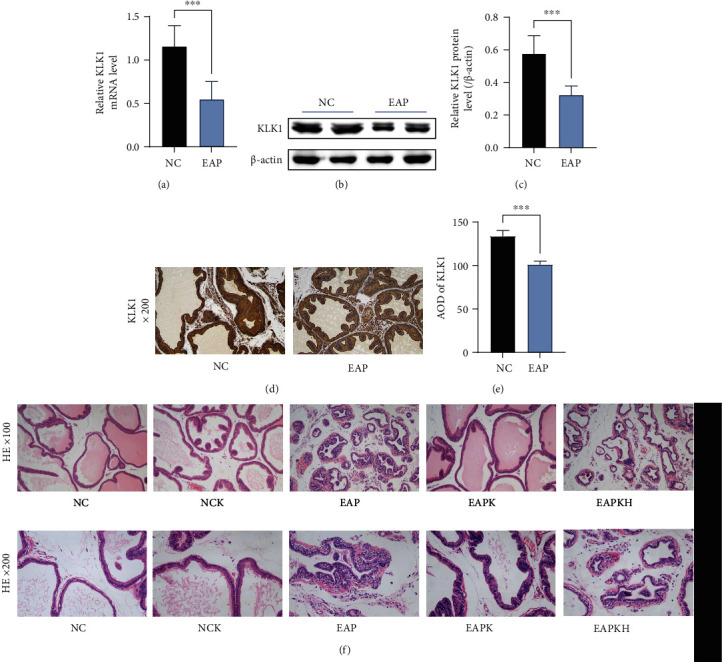
Suppressed expression of KLK1 in the prostate with inflammation and the conventional pathological manifestations of rat prostates. (a) The relative mRNA expression level of KLK1 to *β*-actin by QRT-PCR. (b) Representative western blot results of KLK1 protein expression. *β*-Actin served as an internal control. (c) The relative optical density quantification of KLK1 according to western blot. (d) KLK1 protein expression and location in the prostate by IHC (magnification ×200). (e) The AOD quantification of KLK1 according to IHC. Each bar in (c), (d), and (e) represents the mean ± SD. ^∗∗∗^*P* < 0.001; ^∗∗∗∗^*P* < 0.0001. (f) Representative H&E photos of the prostate samples of the rats (upper, magnification ×100; lower, magnification ×200).

Through the results of H&E sections, we confirmed that the EAP group was successfully induced with chronic prostatitis, which manifested as gland atrophy, thickened smooth muscle, and diffuse infiltration of inflammatory cells (Figure 2(f)). Moreover, compared with the NC group, the mRNA and protein expression levels of KLK1 in the prostates of the EAP group were significantly reduced (Figures 2(a)–2(c)). It could be seen from the IHC photos that the expression of KLK1 in prostate epithelial cells in the EAP group was lower than that in the NC group (Figures 2(d) and 2(e)). These results suggested that the expression of KLK1 in the prostate was inhibited during the development of chronic prostatitis. However, injection of KLK1 could reduce inflammatory damage, which manifested as normal glandular epithelium and glandular cavity, and significantly reduced inflammatory cell infiltration in the prostates of the EAPK group (Figure 2(f)). Notably, KLK1 administration to normal rats (NCK group) did not affect their prostate pathological performance and that HOE140 blocking the KLK1/BK signaling pathway (EAPKH group) made the anti-inflammatory effect of KLK1 on prostatitis be canceled (Figure 2(f)).

### 3.2. KLK1 Administration Significantly Suppressed Inflammation of the Prostate

**Figure 3:**
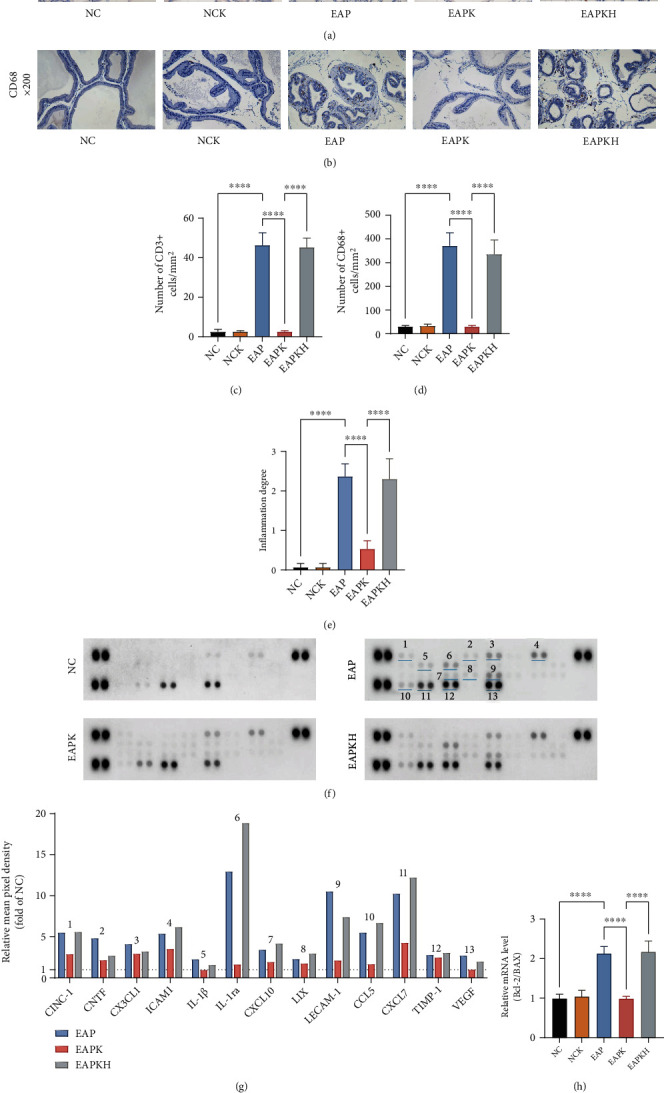
KLK1 inhibited inflammatory damage of the prostate, but HOE140 canceled the beneficial effects of KLK1. (a, b) The representative IHC photos of CD3 and CD68, respectively. (c, d) The counting results of CD3-positive T cells and CD68-positive macrophages according to IHC, respectively. (e) The analysis result of inflammation degrees according to H&E. (f) Developed X-ray films of the Rat Cytokine Array Panel. The cytokines represented by these spots are shown in (g) via the marked numbers. (g) Pixel densities of 13 cytokines with visible differences (the ordinate represents the fold of the NC group). (h) The ratio of the mRNA expression level of Bcl-2 to BAX by QRT-PCR. Each bar in (c), (d), (e), and (h) represents the mean ± SD. ^∗∗∗∗^*P* < 0.0001.

Through ICH photos and cell counts, we found that CD3-positive T cells and CD68-positive macrophages in the EAP group were significantly increased compared to the NC group, but CD3-positive T cells and CD68-positive macrophages in the EAPK group were significantly reduced compared to the EAP group (Figures 3(a)–3(d)). Assessment of inflammation degree also showed that KLK1 administration (EAPK group) significantly reduced the severity of prostatitis (Figure 3(e)). Meanwhile, comparing the NC group with the NCK group, KLK1 administration to normal rats did not cause inflammatory cell infiltration in the prostate. The results of the Rat Cytokine Array Panel showed that CINC-1, CNTF, CX3CL1, ICAM1, IL-1*β*, IL-1ra, CXCL10, LIX, LECAM-1, CCL5, CXCL7, TIMP-1, and VEGF were upregulated in the EAP group (Figures 3(f) and 3(g)). In particular, the increase in IL-1ra, LECAM-1, and CXCL7 of the EAP group exceeded 10 times than the NC group. However, KLK1 administration could reduce the upregulation of all 13 cytokines except TIMP-1. On the other hand, Bcl-2/BAX results showed decreased apoptosis in the EAP group, but the EAPK group had a similar level of apoptosis as the NC group (Figure 3(h)). From the comparison between the EAPKH group and the EAPK group, it could be seen that the effects of KLK1 on reducing inflammatory cell infiltration, inhibiting the production of cytokines, and restoring normal levels of apoptosis were all canceled by HOE140 (Figures 3(a)–3(h)).

### 3.3. KLK1 Administration Inhibited Prostatic Stroma Fibrosis in Chronic Prostatitis

**Figure 4:**
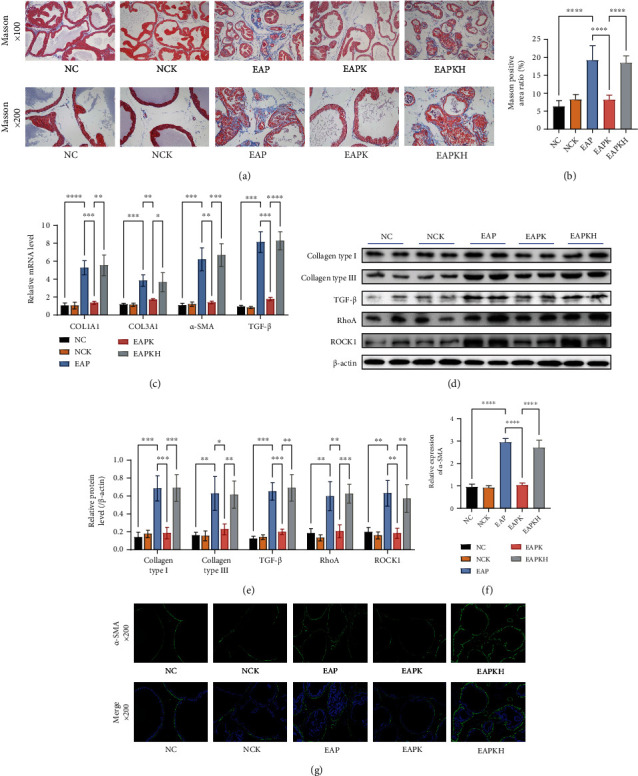
Fibrosis manifestations of the rat prostate and the signal pathway of fibroblasts transdifferentiation into myofibroblasts. (a) Representative Masson photos of the prostate samples of the rats (upper, magnification ×100; lower, magnification ×200). (b) The positive area ratio of Masson staining. (c) Relative mRNA expression levels of COL1A1, COL3A1, *α*-SMA, and TGF-*β* to *β*-actin by QRT-PCR. (d) Representative western blot results of Collagen Type I, Collagen Type III, TGF-*β*, RhoA, and ROCK1 protein expressions. *β*-Actin served as an internal control. (e) The relative optical density quantification of the proteins mentioned above according to western blot. (f) The fluorescence intensity of *α*-SMA. (g) Representative IF photos of *α*-SMA of the prostate samples of the rats (upper, green, simple *α*-SMA staining, magnification ×200; lower, merge of *α*-SMA and DAPI, magnification ×200). Each bar in (b), (c), (e), and (f) represents the mean ± SD. ^∗^*P* < 0.05, ^∗∗^*P* < 0.01, ^∗∗∗^*P* < 0.001, and ^∗∗∗∗^*P* < 0.0001.

By Masson's trichrome staining, the area ratio of collagen in the EAP group and EAPKH group was higher than that in the NC group and EAPK group (Figures 4(a) and 4(b)). By QRT-PCR and western blot, the expression levels of Collagen Type I and Collagen Type III in the EAP group and EAPKH group were higher than that in the NC group and EAPK group (Figures 4(c)–4(e)). These results indicated that inflammation induced significant prostate extracellular matrix deposition, but KLK1 administration inhibited this process. Furthermore, by detecting the TGF-*β*/RhoA/ROCK1/*α*-SMA signaling pathway, we found that there was an upregulated process of transdifferentiation from fibroblasts to myofibroblasts in the EAP group (Figures 4(c)–4(g)), but KLK1 administration inhibited this signaling pathway. Labeled by *α*-SMA, myofibroblasts and smooth muscle cells in the EAP group and EAPKH increased significantly (Figures 4(f) and 4(g)). In addition, from all the results (Figures 4(a)–4(g)), comparing the NC group and NCK group, we could find that the administration of KLK1 to normal rats did not lead to prostate fibrosis. And comparing the EAPK group and EAPKH group, HOE140 canceled the effects of KLK1 on antifibrosis and antitransdifferentiation.

### 3.4. KLK1 Ameliorated Prostate Hypoxia, Angiogenesis, and Oxidative Stress Caused by Inflammation

**Figure 5:**
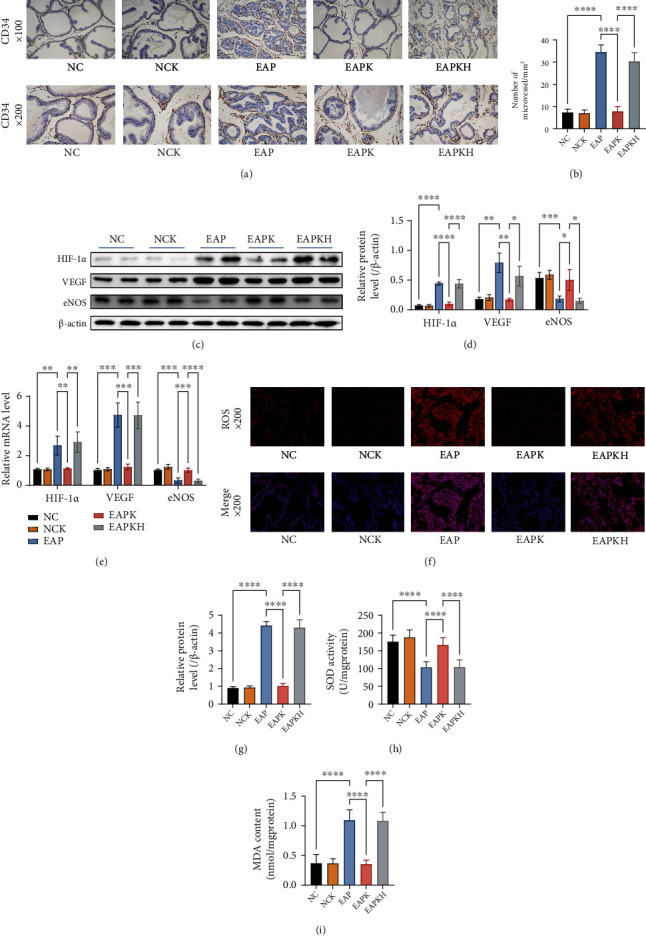
Manifestations of hypoxia, angiogenesis, and oxidative stress in the rat prostate. (a) Representative IHC photos of CD34 of the rat prostate samples (upper, magnification ×100; lower, magnification ×200). (b) The counting result of MVD. (c) Representative western blot results of HIF-1*α*, VEGF, and eNOS protein expressions. *β*-Actin served as an internal control. (d) The relative optical density quantification of the proteins mentioned above according to western blot. (e) Relative mRNA expression levels of HIF-1*α*, VEGF, and eNOS to *β*-actin by QRT-PCR. (f) Representative fluorescence photos of ROS in situ of the prostate samples of the rats (upper, red, simple ROS staining, magnification ×200; lower, merge of ROS and DAPI, magnification ×200). (g) The fluorescence intensity of ROS. (h) SOD activity of the rat prostates. (i) MDA content of the rat prostates. Each bar in (b), (d), (e), (g), (h), and (i) represents the mean ± SD. ^∗^*P* < 0.05, ^∗∗^*P* < 0.01, ^∗∗∗^*P* < 0.001, and ^∗∗∗∗^*P* < 0.0001.

The microvessels were labeled by CD34. After excluding the false positive area of staining around the glands, the counting result showed that the MVD of the EAP group was significantly higher than that of the NC group and the MVD of the EAPK group was significantly lower than that of the EAP group (Figures 5(a) and 5(b)). By QRT-PCR and western blot, the upregulated HIF-1*α* indicated that there was hypoxia in the rat prostates in the EAP group, and the downregulated HIF-1*α* in the EAPK group indicated that the administration of KLK1 significantly ameliorated the hypoxia induced by inflammation (Figures 5(c)–5(e)). Different from the Rat Cytokine Array Panel experiment which only detected one sample in each group, we further detected VEGF expression in all rats by QRT-PCR and western blot. The significantly upregulated VEGF expression level explained the increased MVD in the EAP group; however, KLK1 administration significantly inhibited the expression of VEGF and thus also inhibited angiogenesis (Figures 5(c)–5(e)). Furthermore, inflammation induced rat prostate microvascular dysfunction in the EAP group; however, in the EAPK group, KLK1 administration upregulated eNOS which exerted a protective effect of microvessel (Figures 5(c)–5(e)). ROS in situ fluorescence photos showed that inflammation caused the increase of ROS in the rat prostates of the EAP group, and KLK1 administration could inhibit the production of ROS induced by inflammation (Figures 5(f) and 5(g)). Furthermore, the EAP group showed a significant decrease in SOD activity and a significant increase in MDA content, which meant that inflammation had destroyed the oxidation-antioxidant balance in the prostate and has caused oxidative damage to the prostate (Figures 5(h) and 5(i)). KLK1 administration significantly ameliorated inflammation-induced oxidative stress in the EAPK group, but the results of the EAPKH group showed that HOE140 abolished this effect (Figures 5(h) and 5(i)).

### 3.5. Inflammation Suppressed KLK1 Expression in BPH Patients

**Figure 6:**
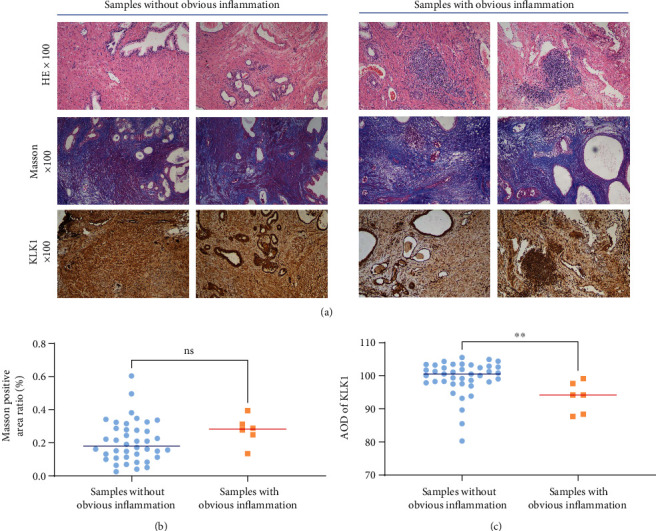
The effect of inflammation on the expression of KLK1 and fibrosis in the prostate of BPH patients. (a) Representative H&E photos, Masson photos, and KLK1 IHC photos of the prostate tissue from patients undergoing BPH surgery (upper, H&E, magnification ×100; middle, Masson, magnification ×100; and lower, IHC, magnification ×100). (b) Statistical analysis of the positive area ratio of Masson staining. ns, *P* > 0.05. (c) Statistical analysis of the AOD quantification of KLK1 according to IHC. ^∗∗^*P* < 0.01.

By examining the HE-stained sections one by one, we found 6 samples with obvious inflammatory infiltration, which had multiple severe inflammatory foci (dense, large-area accumulation of inflammatory cells, as shown in. The samples infiltrated with only a single mild inflammatory focus or a small number of scattered inflammatory cells were assigned to samples without obvious inflammation group. The KLK1 expression level of samples with obvious inflammation was significantly lower than that of samples without obvious inflammation (Figures 6(a) and 6(c)). The level of fibrosis in samples with obvious inflammatory seemed to be higher than that of samples without obvious inflammation, but the results were not statistically significant.

## 4. Discussion

Following the previous study that found the potential protective effect of KLK1 on aged prostate [2], we further explored the role of KLK1 in inflammatory prostate damage in this research. We found that the expression of KLK1 decreased in the inflamed prostate of rats, and KLK1 administration significantly inhibited inflammatory cell infiltration and reduced the production of inflammatory cytokines. Prostate samples from the chronic prostatitis rat model showed increased infiltration of T cells and macrophages, as well as pathological manifestations of inflammatory damage such as gland atrophy, stroma remodeling, collagen deposition, and angiogenesis. KLK1 administration inhibited TGF-*β*-related fibrosis signaling pathways, increased the expression of eNOS as well as ameliorated hypoxia and oxidative stress, and suppressed the proangiogenic VEGF in prostatitis rats. However, after HOE140 blocked BDKRB2, the beneficial effects of KLK1 were all canceled. In addition, KLK1 intervention in normal rats had no obvious side effects. Experimental results on surgical samples of human BPH showed that the expression of KLK1 in the prostate was decreased when BPH was accompanied by obviously serious inflammation. These results suggest that KLK1 or the dysregulation of the kallikrein-kinin system may play an important role in which prostatic inflammation persists and even progresses to BPH.

Ever since it was reported that inflammation was a common finding in histologic prostate specimens, many studies on the pathogenesis of BPH have suggested a role of inflammation in the development of histologic BPH [27, 28]. There might be an autoimmune component in BPH, whereby antigenic stimuli could result in the development of a chronic inflammatory response leading to tissue rebuilding and stromal growth in the prostate [29]. We previously reported that mild chronic autoimmune prostatitis would evolve into BPH assessed by a cumulative pathology score in a rat model [12]. In our current study, we have chosen a serious autoimmune inflammation induction program in order to show the effect of KLK1 on inflammation more obviously. It should be pointed out that in addition to this model, there are other induction methods of chronic prostatitis, such as the method of intraprostatic injection of 3% *λ*-carrageenan, and usually, they have similar cytokine profiles, such as elevated IL-1 and IL-6 [30, 31]. In this study, we utilized a commercial Rat Cytokine Array Panel. We reported significantly upregulated IL-1 in the experimental result section, but not IL-6. The difference in IL-6 is only clearly visible after the gamma value of the original photo has been adjusted, and we are concerned about the deviations magnified in the adjusted photo, so no differences in IL-6 were reported. However, in our and others' previous similar studies [12, 32], IL-6 was upregulated in the EAP model, so we believe that in this study, the induction of the EAP model was successful, but some inflammatory factors may require more appropriate detection methods to show the significant differences. In this rat EAP model, the subcutaneously injected prostatic antigen continued stimulating the immune system against their own prostate, and infiltrating inflammatory cells chronically damaged the resident cells and vessels of the prostate. The efficiency of oxygen supply to the prostate was poor due to the highly dysregulated peripheral microvasculature. Moreover, this was along with the increased oxygen demands of activated infiltrating inflammatory cells and prostatic cells, promoting a hypoxic microenvironment and mitochondrial dysfunction. Local hypoxia is a further stimulus for the production of inflammatory mediators and growth factors, which forms a vicious circle. By detecting increased HIF-1*α* and ROS, we proved that there were obvious hypoxia and oxidative stress in the rat prostate with inflammation. In addition, the downregulated SOD and the increased MDA indicate that the oxidation-antioxidant balance in this severe prostatitis model has been completely out of balance. Just like that hypoxia and angiogenesis are common findings in human BPH tissue [33, 34], our EAP model successfully simulated BPH-related pathological conditions such as chronic inflammatory cell infiltration, hypoxia, and angiogenesis. On the other hand, it has been reported that reduced apoptosis often occurs in inflamed atrophic lesions [35, 36], which may involve the potential carcinogenic effects of chronic or recurrent inflammation [18]. Although there is no evidence that human BPH will progress to prostate cancer, a reduction in apoptosis in the EAP rat prostates of this study was observed, and KLK1 administration restored the level of apoptosis of them. In fact, due to many factors such as anatomical characteristics and disease course, it is impossible for the rat EAP model to completely simulate the progress of human BPH, but in this study, that KLK1 improved the various inflammatory damages including reduced apoptosis of the EAP model is indeed gratifying. However, only using the EAP model is also one of the limitations of this study. The EAP model can only simulate chronic nonbacterial prostatitis. It is still unknown whether other common types of prostatitis, such as acute prostatitis or bacterial prostatitis, will develop like the above process or be ameliorated by KLK1. Based on the gratifying experimental results in the EAP model, the effect of KLK1 on other prostate models seems to be anticipated. However, different inflammations develop differently, and the anti-inflammatory effect of KLK1 is not directly affecting the inflammatory cells; therefore, the question needs to be answered by another systematic research.

Endothelial dysfunction, which is a common pathophysiological process of aging, hypertension, diabetes, hyperlipidemia, and other clinical risk factors for BPH/LUTS, leads to a vicious cycle of hypoxia, vasoconstriction, altered smooth muscle contractility, and degeneration of autonomic neurons and ganglia [37]. Endothelial dysfunction stems primarily from impaired NO bioavailability and is characterized by a reduced vasodilatory response to endothelial stimuli [37]. Impaired bioavailability of NO can result from reduced eNOS activity or increased breakdown of NO by ROS [37]. Endothelial dysfunction as a critical and common initial step in the pathogenesis of BPH/LUTS could be an excellent therapeutic target. Studies on PDE5I have shown that the upregulated NO/cGMP signaling pathway via inhibition of PDE5 effectively improves endothelial function, resulting in an anti-inflammatory effect on endothelial cells and improvement of prostatic ischemia and fibrosis [38–40]. Considering that KLK1 upregulated the eNOS expression via a BDKRB2-dependent mechanism, we believe that in a similar way to PDE5I, with long-term administration of KLK1, the eNOS/NO/cGMP signal pathway of the prostatic microcirculation could be restored, and then, the endothelial dysfunction and inflammation progression are controlled.

It has been demonstrated that KLK1 through the BDKRB2 signaling pathway exhibits a wide spectrum of beneficial effects [19]. Specifically, KLK1 is expressed in various organs, such as the pancreas, salivary gland, kidney, heart, and prostate, and KLK1 is synthesized as a precursor and converted into the active form by the cleavage of an amino-terminal peptide [41]. BK, the easily degraded vasoactive kinins produced by KLK1 processing LMWK, can bind to BDKRB2 on vascular endothelial cells and activate the eNOS/NO pathway. BDKRB2 is a highly selective, G protein-coupled receptor constitutively expressed in a wide variety of tissues and mediates most of the biological actions of BK [42]. In detail, activation of BDKRB2 stimulates the membrane phospholipid metabolism by activating PLC and promotes the release of NO through an eNOS-dependent mechanism [41]. NO rapidly diffuses across the cell membrane to the cytoplasm of adjacent vascular smooth muscle cells, where it binds and activates sGC which acts on GTP to produce cGMP [43]. Ultimately, PKG phosphorylated by cGMP performs various physiological functions [43]. It has been reported that the expressions of eNOS and cGMP levels in the corpus cavernosum of EAP rats were significantly downregulated [44], and a case-control study demonstrated that men with chronic prostatitis had evidence of increased arterial stiffness and vascular endothelial dysfunction [45]. Through increased production of inflammatory cytokines and cellular adhesion molecules, the dysfunctional endothelium promotes inflammation within the vascular wall, forming a vicious circle [46]. However, in our study, the KLK1 administration blocked this vicious circle. Amplified NO/cGMP pathway could cause the relaxation of vascular smooth muscle via downregulation of intracellular Ca^2+^ level by PKG and, finally, increase the blood supply of the prostate. In this study, we found that KLK1 administration could upregulate eNOS and downregulate HIF-1*α* and VEGF expressions in EAP rats, which meant that prostate endothelial function was restored and hypoxia and angiogenesis were suppressed. After prostate hypoxia and endothelial dysfunction were ameliorated by KLK1, the inflammation-hypoxia vicious cycle was interrupted.

Prostate fibrosis is one of the obvious damages to the prostate caused by chronic inflammation. By the stereological analysis, the patients with symptomatic BPH have a higher stroma-to-epithelium ratio than asymptomatic patients [47]. Prostatic tissue from chronic prostatitis and chronic pelvic ischemic rats showed an increased expression of *α*-SMA and increased deposition of collagen [12, 38]. Infiltrating inflammatory cells and prostate stromal cells affected by inflammation or hypoxia could upregulate the secretion of cytokines which promote the growth and remodeling of prostate stromal [18, 48]. Myofibroblasts transdifferentiated from fibroblasts can secrete a large amount of extracellular matrix, which is an important cause of prostate fibrosis [49]. Increased ROS generated by NOX4 in inflammatory and hypoxic prostate promotes TGF-*β* expression [49], which upregulates the activation of the RhoA/ROCK1 pathway to promote cytoskeletal rearrangement inducing the pathological process characterized by fibroblast-to-myofibroblast transdifferentiation [50]. Our experimental results showed that KLK1 administration downregulated the ROS and TGF-*β*/RhoA/ROCK1 signaling pathways in the EAP rat prostate, thus inhibiting the formation of myofibroblasts and collagen deposition. In our previous research, we found that BK could inhibit the expression of TGF-*β* in prostate stromal cells *in vitro* [2], but in the current study, we believe the main reason for antifibrosis is that KLK1 improved the microcirculation to inhibit hypoxia and the subsequent activation of the TGF-*β*/RhoA/ROCK1 signaling pathway. However, in the long duration of human BPH development, the progression of fibrosis is very complex, and inflammation cannot be the only induction factor. From the experimental results of human specimens, it can be found that the level of fibrosis is higher in the samples with obvious inflammatory than the samples without obvious inflammatory, but there is no statistically significant difference. In our previous study, we also found that the expression level of KLK1 was negatively correlated with the level of prostate fibrosis in human BPH specimens, but there was also no statistically significant difference [2]. Therefore, our current experimental results indeed suggest that KLK1 can alleviate inflammation and thereby reduce prostate fibrosis, but in the complex process of human BPH development, this kind of help is limited, and other mechanisms need to be combined to treat BPH.

Although in this research KLK1 significantly inhibited inflammatory damages in rat prostate, there still seems to be a puzzling relationship between KLK1 and inflammation. Previously, it was generally thought that BK was linked with the pathophysiological processes accompanied with tissue damage and inflammation, because BK and its receptors participate in increasing vascular permeability, venoconstriction, and arterial dilatation; moreover, BK is a potent endogenous algogenic substance [51–53]. Even BK has been experimentally proven to elicit contractile responses in isolated canine prostate tissue and promote the proliferation of primary cultures of normal human prostate stromal cells [54, 55]. In our current study, BK, the downstream active substance of KLK1, played an anti-inflammatory rather than proinflammatory effect after activating BDKRB2. This might be because BDKRB2 was activated mainly in vascular endothelial cells rather than glandular epithelial or stromal cells of the prostate. KLK1 was administered by tail vein injection, and normally, only LMWK in the blood was lysed to produce BK, which is bound to BDKRB2 on the vascular endothelium locally. Importantly, the normal rats injected with KLK1 did not develop prostatic inflammation, indicating that KLK1 is not a potential proinflammatory factor in our study. And whether in BPH patients or EAP rats, the expression of KLK1 in the inflamed prostate is reduced, which also implies that supplementing KLK1 may be exactly what is needed to fight inflammation. In addition, it has been demonstrated that the prostate has a high level of ACE activity, which catalyzes the enzymatic degradation of BK [56, 57]. Therefore, we believe that in this study, exogenous KLK1 and activated BK mainly played a role in the microcirculation of the rat prostate but did not significantly affect the prostate cells directly. On the other hand, we also conducted a preexperiment to study the effect of KLK1 administration at different periods on chronic prostatitis (the experimental design and specific results of the preexperiment are shown in Supplemental File 1). In the preexperiment, we compared the results of KLK1 administration at the same time as the EAP model building and KLK1 administration after the EAP model was completed. The KLK1 administration at the same time as the model building could better exert the therapeutic effect of KLK1, which was specifically manifested in less inflammatory cell infiltration and fewer inflamed atrophic lesions. This means that the anti-inflammatory mechanism of KLK1 is mainly in the early stage of inflammation, and if the inflammation has become chronic, the therapeutic effect of KLK1 would be diluted. Based on this, we have further conjectures about the role of KLK1 against autoimmune prostatitis. In the process of autoimmune inflammation induction, the antigen injected subcutaneously first triggers an immune response to the prostate. After inflammatory cells attack and destroy prostate cells, more prostate antigens would be exposed to aggravate the immune response. If KLK1 administration and improvement of microcirculation dysfunction occurred at the beginning of the induction process, the local prostatic inflammatory damage could be controlled by some homeostatic mechanisms, so that there would be no more generation of prostate antigen to reinforce the immune response later. On the contrary, if the inflammatory cells have caused significant damage to the prostate parenchyma, even if KLK1 restored the prostate's microcirculation function, it is difficult for the body to completely stop the chronic inflammatory damage through self-regulation.

Finally, although we analyzed that KLK1 mainly played a protective effect on inflamed prostate by improving microcirculation dysfunction, we did not conduct more detailed cell experiments to prove it. The experiments to prove the mechanism may involve the construction of a more complicated system of vascular endothelial cells, inflammatory cells, and prostate cells *in vitro*. The subsequent in-depth study of inflamed prostate should analyze the specific types of inflammatory cells regulated by KLK1/BK/BDKRB2/eNOS/NO and their roles in microcirculation dysfunction, as well as the unclear homeostatic self-regulation mechanism. On the other hand, the results of this study also show us some prospects. The RhoA/ROCK1 signaling pathway, which has been proven to be affected by KLK1 in the prostate, is also closely related to the contraction and tone regulation of the bladder [58]. And autonomic/sensory nerve dysfunction caused by endothelial dysfunction is also involved in the abnormal sensation and activity of the bladder [37]. Therefore, the effect of KLK1 on the bladder suffering from BPH/LUTS is also worth exploring.

## 5. Conclusions

The KLK1 expression is reduced in the inflamed prostate. Intravenous administration of exogenous KLK1 can reduce the inflammatory infiltration of the rat prostate and inhibit chronic inflammatory damage, including hypoxia, oxidative stress, angiogenesis, and fibrosis via ameliorating endothelial dysfunction. The BDKRB2-dependent endothelial protection mechanism plays an important role in the prostate and deserves further study.

## Data Availability

The data of the materials and methods and results to support the conclusions are included in this article. If any other data are needed, please contact the corresponding author.

## Abbreviations

AOD: Average optical density
*α*-SMA: *α*-Smooth muscle actin
BDKRB2: Bradykinin receptor B2
BK: Bradykinin
BPH: Benign prostate hyperplasia
BSA: Bovine serum albumin
cGMP: Cyclic guanosine monophosphate
EAP: Experimental autoimmune prostatitis
eNOS: Endothelial nitric oxide synthase
GTP: Guanosine triphosphate
H&E: Hematoxylin-eosin
HIF-1*α*: Hypoxia inducible factor-1*α*
HRP: Horseradish peroxidase
IF: Immune-fluorescence
IHC: Immune-histochemistry
KKS: Tissue kallikrein-kinin system
KLK1: Tissue kallikrein
LMWK: Low molecular weight kininogen
LUTS: Lower urinary tract symptoms
MDA: Malondialdehyde
MVD: Microvessel density
NOX4: NADPH oxidase 4
NS: Normal saline
PKG: cGMP-dependent protein kinase
PLC: Phospholipase C
PDE5I: Phosphodiesterase 5 inhibitor
PVDF: Polyvinylidene fluoride
QRT-PCR: Quantitative real-time polymerase chain reaction
RhoA: Ras homolog family member A
ROCK1: Rho-associated coiled-coil containing protein kinase 1
ROS: Reactive oxygen species
SD: Standard deviation
SDS: Sodium dodecyl sulfate
sGC: Soluble guanylate cyclase
SOD: Superoxide dismutase
SPF: Specific pathogen free
TGF-*β*: Transforming growth factor *β*1
VEGF: Vascular endothelial growth factor.

## Supplementary Materials

Supplementary Materials The experimental design and results of the preexperiment investigating the effects of KLK1 administration at different periods on chronic prostatitis are presented in Supplementary File 1.

## References

[1] Gratzke C., Bachmann A., Descazeaud A. (2015). EAU guidelines on the assessment of non-neurogenic male lower urinary tract symptoms including benign prostatic obstruction. *European Urology*.

[2] Zhang M., Luo C., Lin D., Cui K., Chen Z., Liu J. (2021). Human tissue kallikrein 1 is downregulated in elderly human prostates and possesses potential in vitro antioxidative and antifibrotic effects in rodent prostates. *Oxidative Medicine and Cellular Longevity*.

[3] De Nunzio C., Presicce F., Tubaro A. (2016). Inflammatory mediators in the development and progression of benign prostatic hyperplasia. *Nature Reviews. Urology*.

[4] Gandaglia G., Briganti A., Gontero P. (2013). The role of chronic prostatic inflammation in the pathogenesis and progression of benign prostatic hyperplasia (BPH). *BJU International*.

[5] Fibbi B., Penna G., Morelli A., Adorini L., Maggi M. (2010). Chronic inflammation in the pathogenesis of benign prostatic hyperplasia. *International Journal of Andrology*.

[6] Tong Y., Zhou R. Y. (2020). Review of the roles and interaction of androgen and inflammation in benign prostatic hyperplasia. *Mediators of Inflammation*.

[7] Tsunemori H., Sugimoto M. (2021). Effects of inflammatory prostatitis on the development and progression of benign prostatic hyperplasia: a literature review. *International Journal of Urology*.

[8] Nickel J. C., Roehrborn C. G., O'Leary M. P., Bostwick D. G., Somerville M. C., Rittmaster R. S. (2008). The relationship between prostate inflammation and lower urinary tract symptoms: examination of baseline data from the REDUCE trial. *European Urology*.

[9] Delongchamps N. B., De La Roza G., Chandan V. (2008). Evaluation of prostatitis in autopsied prostates--is chronic inflammation more associated with benign prostatic hyperplasia or cancer?. *The Journal of Urology*.

[10] Franiel T., Ludemann L., Rudolph B. (2009). Prostate MR imaging: tissue characterization with pharmacokinetic volume and blood flow parameters and correlation with histologic parameters. *Radiology*.

[11] Scott Lucia M., Lambert J. R. (2008). Growth factors in benign prostatic hyperplasia: basic science implications. *Current Urology Reports*.

[12] Zhang M., Luo C., Cui K., Xiong T., Chen Z. (2020). Chronic inflammation promotes proliferation in the prostatic stroma in rats with experimental autoimmune prostatitis: study for a novel method of inducing benign prostatic hyperplasia in a rat model. *World Journal of Urology*.

[13] Kamijo T., Sato S., Kitamura T. (2001). Effect of cernitin pollen-extract on experimental nonbacterial prostatitis in rats. *Prostate*.

[14] Wong L., Hutson P. R., Bushman W. (2014). Prostatic inflammation induces fibrosis in a mouse model of chronic bacterial infection. *PLoS One*.

[15] Wong L., Hutson P. R., Bushman W. (2015). Resolution of chronic bacterial-induced prostatic inflammation reverses established fibrosis. *Prostate*.

[16] Okamoto K., Kurita M., Yamaguchi H., Numakura Y., Oka M. (2018). Effect of tadalafil on chronic pelvic pain and prostatic inflammation in a rat model of experimental autoimmune prostatitis. *Prostate*.

[17] Sugimoto M., Zhang X., Ueda N. (2019). A phosphodiesterase 5 inhibitor, tadalafil, suppresses stromal predominance and inflammation in a rat model of nonbacterial prostatitis. *BMC Urology*.

[18] Lucia M. S., Torkko K. C. (2004). Inflammation as a target for prostate cancer chemoprevention: pathological and laboratory rationale. *The Journal of Urology*.

[19] Chao J., Shen B., Gao L., Xia C. F., Bledsoe G., Chao L. (2010). Tissue kallikrein in cardiovascular, cerebrovascular and renal diseases and skin wound healing. *Biological Chemistry*.

[20] Devetzi M., Goulielmaki M., Khoury N. (2018). Genetically‑modified stem cells in treatment of human diseases: tissue kallikrein (KLK1)‑based targeted therapy (review). *International Journal of Molecular Medicine*.

[21] Alexander-Curtis M., Pauls R., Chao J., Volpi J. J., Bath P. M., Verdoorn T. A. (2019). Human tissue kallikrein in the treatment of acute ischemic stroke. *Therapeutic Advances in Neurological Disorders*.

[22] Cui K., Luan Y., Wang T. (2017). Reduced corporal fibrosis to protect erectile function by inhibiting the Rho-kinase/LIM-kinase/cofilin pathway in the aged transgenic rat harboring human tissue kallikrein 1. *Asian Journal of Andrology*.

[23] Cui K., Luan Y., Tang Z. (2017). Involvement of DDAH/ADMA/NOS/cGMP and COX-2/PTGIS/cAMP pathways in human tissue kallikrein 1 protecting erectile function in aged rats. *PLoS One*.

[24] Cui K., Luan Y., Tang Z. (2019). Human tissue kallikrein-1 protects against the development of erectile dysfunction in a rat model of hyperhomocysteinemia. *Asian Journal of Andrology*.

[25] Hock F. J., Wirth K., Albus U. (1991). Hoe 140 a new potent and long acting bradykinin-antagonist: in vitro studies. *British Journal of Pharmacology*.

[26] Breser M. L., Motrich R. D., Sanchez L. R., Rivero V. E. (2017). Chronic pelvic pain development and prostate inflammation in strains of mice with different susceptibility to experimental autoimmune prostatitis. *Prostate*.

[27] Bostanci Y., Kazzazi A., Momtahen S., Laze J., Djavan B. (2013). Correlation between benign prostatic hyperplasia and inflammation. *Current Opinion in Urology*.

[28] Di Silverio F., Gentile V., De Matteis A. (2003). Distribution of inflammation, pre-malignant lesions, incidental carcinoma in histologically confirmed benign prostatic hyperplasia: a retrospective analysis. *European Urology*.

[29] Kramer G., Mitteregger D., Marberger M. (2007). Is benign prostatic hyperplasia (BPH) an immune inflammatory disease?. *European Urology*.

[30] Šutulović N., Grubač Ž., Šuvakov S. (2021). Experimental chronic prostatitis/chronic pelvic pain syndrome increases anxiety-like behavior: the role of brain oxidative stress, serum corticosterone, and hippocampal parvalbumin-positive interneurons. *Oxidative Medicine and Cellular Longevity*.

[31] Šutulović N., Grubač Ž., Šuvakov S. (2019). Chronic prostatitis/chronic pelvic pain syndrome increases susceptibility to seizures in rats and alters brain levels of IL-1*β* and IL-6. *Epilepsy Research*.

[32] Liu Y., Wazir J., Tang M. (2021). Experimental autoimmune prostatitis: different antigens induction and antigen-specific therapy. *International Urology and Nephrology*.

[33] Lekas A. G., Lazaris A. C., Chrisofos M. (2006). Finasteride effects on hypoxia and angiogenetic markers in benign prostatic hyperplasia. *Urology*.

[34] Park H., Park S., Kim K. H., Cho M. S., Sung S. H., Ro J. Y. (2014). Stromal nodules in benign prostatic hyperplasia: morphologic and immunohistochemical characteristics. *Prostate*.

[35] De Marzo A. M., Marchi V. L., Epstein J. I., Nelson W. G. (1999). Proliferative inflammatory atrophy of the prostate: implications for prostatic carcinogenesis. *The American Journal of Pathology*.

[36] Park D. S., Shim J. Y. (2008). Histologic influence of doxazosin and finasteride in benign prostatic hyperplasia accompanying chronic inflammation. *Urologia Internationalis*.

[37] Cellek S., Cameron N. E., Cotter M. A., Fry C. H., Ilo D. (2014). Microvascular dysfunction and efficacy of PDE5 inhibitors in BPH-LUTS. *Nature Reviews. Urology*.

[38] Zarifpour M., Nomiya M., Sawada N., Andersson K. E. (2015). Protective effect of tadalafil on the functional and structural changes of the rat ventral prostate caused by chronic pelvic ischemia. *Prostate*.

[39] Kurita M., Yamaguchi H., Okamoto K., Kotera T., Oka M. (2018). Chronic pelvic pain and prostate inflammation in rat experimental autoimmune prostatitis: effect of a single treatment with phosphodiesterase 5 inhibitors on chronic pelvic pain. *Prostate*.

[40] Yoshinaga R., Kawai Y., Oka M., Fuchikami C., Oyama T. (2015). Effect of a single treatment with tadalafil on blood flow in lower urinary tract tissues in rat models of bladder overdistension/emptying and abdominal aorta clamping/release. *European Journal of Pharmacology*.

[41] Dutra R. C. (2017). Kinin receptors: key regulators of autoimmunity. *Autoimmunity Reviews*.

[42] Sriramula S. (2020). Kinin B1 receptor: a target for neuroinflammation in hypertension. *Pharmacological Research*.

[43] Friebe A., Koesling D. (2003). Regulation of nitric oxide-sensitive guanylyl cyclase. *Circulation Research*.

[44] Hu Y., Niu X., Wang G., Huang J., Liu M., Peng B. (2016). Chronic prostatitis/chronic pelvic pain syndrome impairs erectile function through increased endothelial dysfunction, oxidative stress, apoptosis, and corporal fibrosis in a rat model. *Andrology*.

[45] Shoskes D. A., Prots D., Karns J., Horhn J., Shoskes A. C. (2011). Greater endothelial dysfunction and arterial stiffness in men with chronic prostatitis/chronic pelvic pain syndrome--a possible link to cardiovascular disease. *The Journal of Urology*.

[46] Vlachopoulos C., Ioakeimidis N., Rokkas K. (2015). Acute effect of sildenafil on inflammatory markers/mediators in patients with vasculogenic erectile dysfunction. *International Journal of Cardiology*.

[47] Shapiro E., Becich M. J., Hartanto V., Lepor H. (1992). The relative proportion of stromal and epithelial hyperplasia is related to the development of symptomatic benign prostate hyperplasia. *The Journal of Urology*.

[48] Yoo T. K., Cho H. J. (2012). Benign prostatic hyperplasia: from bench to clinic. *Korean Journal of Urology*.

[49] Sampson N., Berger P., Zenzmaier C. (2012). Therapeutic targeting of redox signaling in myofibroblast differentiation and age-related fibrotic disease. *Oxidative Medicine and Cellular Longevity*.

[50] Kardassis D., Murphy C., Fotsis T., Moustakas A., Stournaras C. (2009). Control of transforming growth factor *β* signal transduction by small GTPases. *The FEBS Journal*.

[51] Calixto J. B., Cabrini D. A., Ferreira J., Campos M. M. (2000). Kinins in pain and inflammation. *Pain*.

[52] Couture R., Harrisson M., Vianna R. M., Cloutier F. (2001). Kinin receptors in pain and inflammation. *European Journal of Pharmacology*.

[53] Dray A., Perkins M. (1993). Bradykinin and inflammatory pain. *Trends in Neurosciences*.

[54] Srinivasan D., Kosaka A. H., Daniels D. V., Ford A. P., Bhattacharya A. (2004). Pharmacological and functional characterization of bradykinin B_2_ receptor in human prostate. *European Journal of Pharmacology*.

[55] Steidle C. P., Cohen M. L., Neubauer B. L. (1990). Bradykinin-induced contractions of canine prostate and bladder: effect of angiotensin-converting enzyme inhibition. *The Journal of Urology*.

[56] Yokoyama M., Hiwada K., Kokubu T., Takaha M., Takeuchi M. (1980). Angiotensin-converting enzyme in human prostate. *Clinica Chimica Acta*.

[57] van Sande M. E., Scharpe S. L., Neels H. M., Van Camp K. O. (1985). Distribution of angiotensin converting enzyme in human tissues. *Clinica Chimica Acta*.

[58] Morelli A., Filippi S., Sandner P. (2009). Vardenafil modulates bladder contractility through cGMP-mediated inhibition of RhoA/Rho kinase signaling pathway in spontaneously hypertensive rats. *The Journal of Sexual Medicine*.

