# Supplementary material for: Tissue Kallikrein Protects Rat Prostate against the Inflammatory Damage in a Chronic Autoimmune Prostatitis Model via Restoring Endothelial Function in a Bradykinin Receptor B2-Dependent Way
Source: Oxid Med Cell Longev. 2022 Feb 1;2022:1247806. doi: 10.1155/2022/1247806 (PMC8826119; doi:10.1155/2022/1247806)

Pre-Experiment

Because it is uncertain whether KLK1 can ameliorate inflammation, and even it cannot be ruled out that KLK1 may aggravate the progression of inflammation. We designed this pre-experiment before the formal research begins in order to explore the effect of KLK1 on prostatitis preliminarily. The induction method of rat EAP model and the dosage of KLK1 are the same as those in the main article. The specific grouping and administration procedures are shown in the figure1 on page 2. The HE stained photos of rat prostate are shown in the figure2 on page 3.

Figure1. Animal grouping and flowchart of pre-experiment

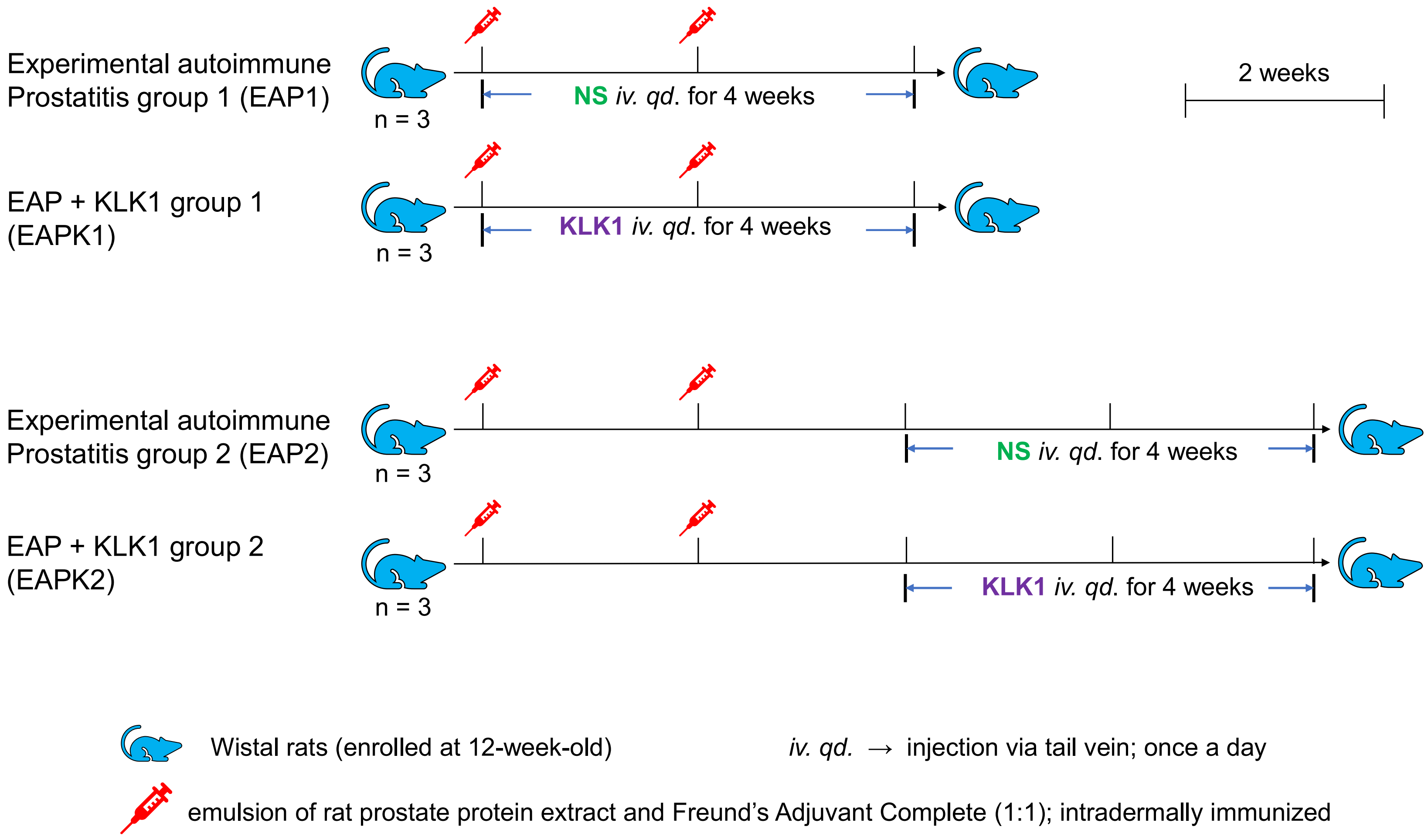

Figure2. Representative HE photos of rats prostate in pre-experiment (magnification ×100)

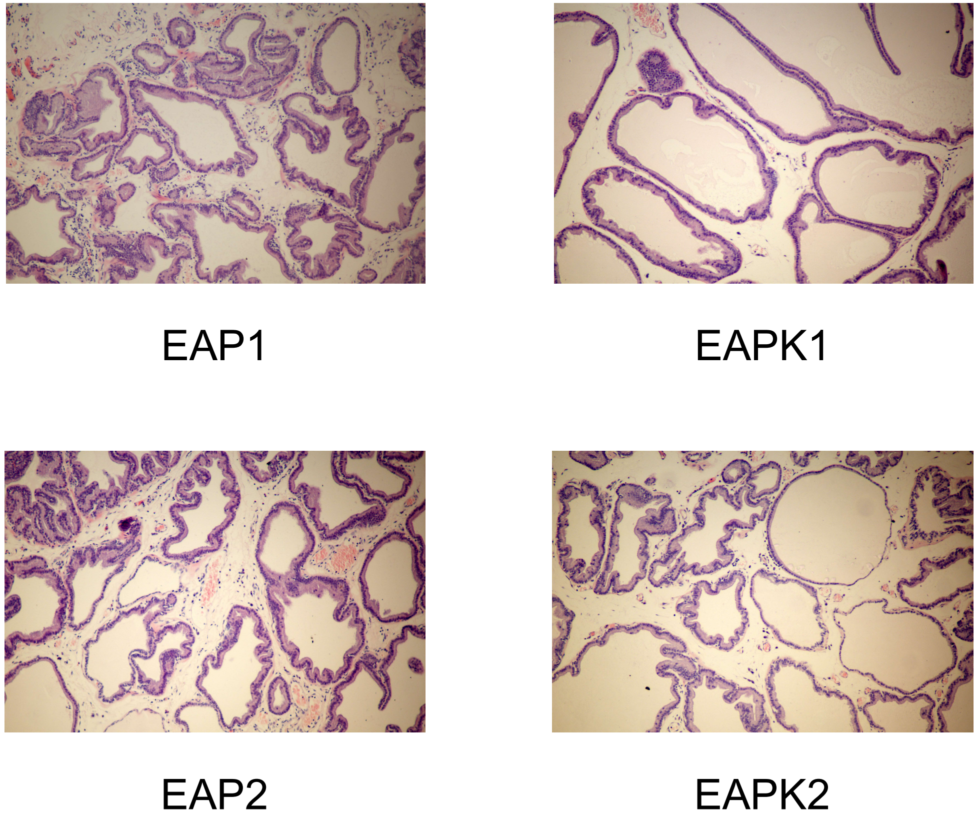

Supplement: Supplementary Materials — The experimental design and results of the preexperiment investigating the effects of KLK1 administration at different periods on chronic prostatitis are presented in Supplementary File 1. [file 1247806.f1.pdf]
